# Maximizing cost savings and clinical outcomes: A retrospective analysis of antimicrobial stewardship for meropenem utilization in Saudi Arabia

**DOI:** 10.1371/journal.pone.0328673

**Published:** 2025-07-17

**Authors:** Rawan T. Tafish, Reem Al-Zayer, Faisal Al-Anazi, Jida Al-Mulki

**Affiliations:** 1 Department of Pharmacy, Specialized Medical Hospitals, Riyadh, Saudi Arabia; 2 Mohammed Al-Mana College for Medical Sciences, Dammam, Saudi Arabia; 3 Department of Epidemiology and Population Health, American University of Beirut, Beirut, Lebanon; Nitte University, INDIA

## Abstract

**Background:**

Meropenem, a key antibiotic for multidrug-resistant infections, is often misused, leading to antimicrobial resistance, increased healthcare costs, and excessive consumption. Optimizing its use through antimicrobial stewardship program is crucial to improving clinical outcomes, reducing resistance, and minimizing unnecessary expenditures.

**Objectives:**

To evaluate trends in meropenem utilization, compliance, consumption, and cost across consecutive quarters from April 2022 to December 2023, highlighting the impact of the gradual implementation of antimicrobial stewardship guidelines and the pivotal role of clinical pharmacists in improving patient outcomes.

**Methods:**

A retrospective observational study was conducted in a tertiary-care private hospital in Saudi Arabia between April 2022 and December 2023. Meropenem utilization was assessed quarterly based on predefined key indicators to evaluate compliance with antimicrobial stewardship guidelines; Q1-2022 served as a baseline for comparison. Consumption was measured in defined daily doses (DDD) per 1,000 patient-days, and cost analysis was performed using Antimicrobial Cost Per Patient Day (ACPD). The main outcome measures included clinical improvement within 72 hours and transfer or discharge in good clinical condition.

**Results:**

Overall compliance with antimicrobial stewardship program guidelines improved significantly from 85.7% in 2022 to 100% in 2023 (p = 0.02). Specific indicators showed substantial progress: compliance with drug interaction considerations increased from 93.8% (123/131) to 98.7% (157/159), appropriate duration compliance rose from 68.3% (82/120) to 90.3% (112/124), and adherence to culture follow-up interventions improved from 51.2% (43/84) to 81.7% (67/82) (all p < 0.05). However, compliance with appropriate dosing declined significantly from 89.3% (117/131) to 82% (123/150). By late 2023, enhanced compliance correlated with better clinical outcomes. Meropenem consumption decreased by 47%, resulting in cumulative savings estimated at 554,285 Saudi Riyals (S.R.), with expenses reduced to approximately 56,365.19 S.R.

**Conclusion:**

The phased implementation of an Antimicrobial Stewardship Program in the clinical setting effectively optimized meropenem use, reducing consumption and costs, and improving prescribing practices and patient outcomes.

## Introduction

Antimicrobial resistance (AMR) is a growing global health crisis with profound public health, economic, and societal consequences. By 2050, AMR is projected to cause 10 million deaths annually and drive healthcare costs up by nearly one trillion US dollars [1]. In addition, all countries around the globe regardless of their economic situation will face the drastic consequences of AMR if proper measures are not employed to contain the crisis [2]. A key driver of AMR is the inappropriate use of broad-spectrum antibiotics, particularly carbapenems, which play an essential role in treating multidrug-resistant infections. Alarmingly, the spread of carbapenem resistance genes has been documented across the Eastern Mediterranean Region, including Saudi Arabia [3–8].

Meropenem, a broad-spectrum carbapenem, exhibits potent bactericidal activity against Gram-negative pathogens while also demonstrating efficacy against Gram-positive bacteria and anaerobes [3]. However, its broad-spectrum nature makes it highly susceptible to misuse, accelerating resistance and undermining its clinical utility [9]. The overuse and inappropriate prescribing of meropenem have been linked to the emergence of carbapenem-resistant pathogens, leading to prolonged hospital stays, increased mortality, and escalating healthcare costs [10,11]. Given these critical consequences, urgent action is needed to optimize meropenem use while ensuring its availability for critically ill patients.

Antimicrobial Stewardship Program (ASP) offers a promising strategy to improve prescribing practices, optimizes antimicrobial utilization, and curbs resistance development [12]. Through interventions such as dose optimization, adherence to culture-guided therapy, and pharmacist-led initiatives, ASPs have demonstrated significant clinical benefits while also reducing healthcare expenditures [13,14]. Studies have shown that ASP implementation is associated with lower rates of hospital-acquired multidrug resistance, shorter treatment durations, and reduced length of hospital stay [15,16]. Additionally, ASPs have been linked to substantial cost savings, with reports indicating a 14% reduction in antimicrobial costs within the first year of implementation [17]. Another study observed a decrease in hospital antimicrobial expenditures from $86,378,300 to $75,028,266 following ASP adoption [18]. In Saudi Arabia, the gradual implementation of ASPs resulted in an average decrease in antimicrobial expenditures by 28%, with cumulative cost savings estimated at S.R. 6,286,929 and minimal additional expenses [19].

Despite the extensive research on the benefits of ASPs in optimizing antibiotic use, data on the real-world impact of ASP implementation on meropenem consumption, cost, compliance, and utilization trends remain limited, particularly in tertiary care settings [20]. The lack of comprehensive insight into meropenem usage patterns hinders the development of effective antibiotic stewardship programs and resistance mitigation strategies [21].

This study aims to evaluate the effectiveness of phased ASP implementation in optimizing meropenem utilization, consumption, and cost at a tertiary hospital in Riyadh, Saudi Arabia. By assessing longitudinal trends in meropenem prescribing and expenditures, this research will help identify key challenges, highlight areas for improvement, and support the development of evidence-based ASP policies to enhance antimicrobial stewardship efforts and promote cost-effective strategies.

## Materials and methods

### Research design and setting

This was a retrospective observational study conducted at SMC hospital, a 160-bed tertiary care hospital in Riyadh, Saudi Arabia. The study included a review of medical records for hospitalized adult and pediatric/neonate patients who received meropenem between April 1, 2022 and December 31, 2023, spanning the second quarter of 2022 through the fourth quarter of 2023. Patients were randomly selected from all wards, including critical care units, inpatient units, long-term units, and surgical units.

Meropenem usage was checked each quarter using a checklist aligned with the hospital’s ASP guideline, developed in concordance with international and local guidelines (IDSA, MOH). Eight key indicators were evaluated: correct indication, absence of hypersensitivity, absence of drug interaction, correct dose, dose adjustment in renal failure, correct duration, culture sensitivity test, and culture follow-up with interventions. The data obtained is presented as a checklist; if the action taken on the patient aligns with the requirement specified in the guideline recommendations, it receives a score of 1; otherwise, the score given is 0; if the indicator is not applicable to meropenem utilization in the tested patient condition, it is referred to as N/A

### Study objectives

The study objectives were:

- To compare trends in meropenem utilization compliance, consumption, cost and utilization across the quarters of 2022 and 2023
- To assess the impact of meropenem utilization compliance on patients’ clinical outcomes
- To determine whether phased ASP interventions led to significant improvements in appropriate prescribing, adherence to guidelines, and cost savings

Inclusion Criteria

- Patients of all ages admitted to any department of the institution between April 2022 and December 2023 who received at least one dose of meropenem were included in the study.

Exclusion Criteria

- Patients prescribed meropenem but did not receive it
- Patients prescribed meropenem in the outpatient clinics

### Recruitment strategy and data collection

The sample size of 291 patients was determined to meet the International Quality Standards, with an average of 41 patients per quarter. The sample size was calculated to achieve a statistical power of at least 80% with a significance level of 5%, ensuring robust statistical analysis and reliable conclusions regarding the appropriateness of meropenem use.

Eligible patients were identified through a review of electronic health records during the study period. Data were accessed for research purposes between July 1 and July 20, 2024. The data collected followed the inclusion criteria to assess the appropriateness of meropenem usage in each quarter using the audit tool, which is in concordance with authorized drug references (i.e., Lexicomp®) and international and local guidelines (IDSA, MOH).

### Clinical pharmacist/infectious disease consultant collaboration

Since the antimicrobial stewardship committee is led by the clinical pharmacist and the infectious disease consultant, restricted antimicrobial agent (including meropenem) prescribing notification reaches them via automatic notification systems awaiting their interventions within 48 hours of prescribing. Moreover, as key members in ASP, routine antimicrobial stewardship rounds have been conducted through the multidisciplinary team (physicians, microbiologists, nurses) to assess and optimize restricted antimicrobial agent utilization, including meropenem.

### Variables assessed

#### Primary outcomes.

1. **Clinical improvement within 72 hours**: This includes a reduction in symptoms such as fever, shortness of breath or inflammation, improvement in vital signs, enhanced physical function or mobility, or positive changes in laboratory results or imaging studies.
2. **Transfer or discharge in good clinical condition**: when the patient is deemed stable and healthy enough to be moved to another facility or sent home.

#### Independent variables.

1. Correct indication: verification of whether the indication for meropenem use was appropriate
2. Absence of hypersensitivity: verification of proper prescribing of meropenem (graded-challenge with monitoring when necessary) and ensuring minimal cross-reactivity in patients with a history of meropenem allergy or beta-lactam allergy, respectively
3. Absence of drug interactions: documentation or intervention for potential drug interactions (i.e., concomitant administration of valproic acid with meropenem), including therapeutic drug monitoring when necessary
4. Correct dose: assessment of whether the administered dose was appropriate
5. Dose adjustment in renal failure: evaluation of whether dose adjustment was required and made in cases of renal failure
6. Correct duration of treatment: evaluation of the duration of meropenem treatment according to treated infection
7. Culture Sensitivity Testing: verification of whether microbial cultures were obtained prior to or during treatment
8. Culture follow-up with interventions: monitoring and intervention based on bacterial culture results (i.e., de-escalation)

#### Secondary outcomes.

Drug consumption, measured in defined daily doses (DDD), was obtained from electronic pharmacy dispensing records. According to WHO Collaborating Centre for Drug Statistics Methodology guidelines, the DDD is defined as the assumed average daily maintenance dose, and is considered as an important indicator for antibiotic use. Antimicrobial use was quantified as DDDs per 1,000 acute patient-days by dividing the total consumed dose (in grams) by the standard daily dose and expressing it per 1,000 patient-days.

The total antimicrobial cost of meropenem was compared across quarters and the current acquisition price was considered for all cost comparisons. The total expenditure of meropenem was calculated and then divided by the total patient-days to calculate the “Antimicrobial Cost Per Patient Day” (ACPD). Q1-2022 was included as a baseline for comparison; cost savings were calculated by subtracting ACPD in each quarter from Q2-2022 to Q4-2023 from Q1-2022 and multiplying the difference by the total patient-days for each year.

#### Phases of implementation.

The improvement in meropenem utilization during the study period (Q2-2022 to Q4-2023) was achieved through a series of strategic phases implemented quarterly. Each phase introduced new strategies aimed at progressively enhancing meropenem prescribing practices. These phases were as follows:

##### Phase 1 (Q2-2022 to Q3-2022):

This phase focused on revitalizing and updating the institution’s antimicrobial stewardship policies and protocols. A dedicated antimicrobial stewardship committee was established with updated terms of reference. Educational sessions and active surveillance were conducted to raise awareness among clinicians. However, the enforcement of policies during this phase was gradual and cautious.

##### Phase 2 (Q3-2022 to Present):

During this phase, meropenem use was restricted, and clinical pharmacist or infectious disease specialist interventions were mandated within 48 hours of prescribing. Dispensing was withheld pending these reviews. Educational sessions and active surveillance continued to reinforce compliance. The implementation of these measures was progressive, allowing clinicians to adapt gradually.

##### Phase 3 (Q2-2023 to Present):

This phase introduced multidisciplinary antimicrobial stewardship rounds in critical care units, which significantly strengthened adherence to ASP guidelines.

### Statistical analysis

Descriptive statistics were employed to summarize ASP indicators: categorical variables were reported as counts and percentages, while continuous variables were presented as means with standard deviations. The overall compliance percentage, calculated as the average of selected ASP indicators, was expressed as the median with interquartile range (IQR) due to its non-normal distribution. Chi-square tests assessed associations between categorical variables, with Fisher’s exact test applied when expected cell counts were below five. The Mann-Whitney U test compared ranks of continuous variables between years for non-parametric data. ANOVA with Bonferroni adjustment compared DDD per 1,000 patient-days across consecutive quarters, while Kruskal-Wallis with Bonferroni adjustment was used for ACPD across quarters. Trends in clinical improvement and early discharge outcomes across seven quarters from 2022 to 2023 were assessed using the Cochran-Armitage trend test. A P-value < 0.05 was considered statistically significant. All analyses were conducted using SPSS version 26, and charts were created using Excel (version 2021; Microsoft Corp, Redmond, WA).

### Ethics approval

This study was performed in line with the principles of the Declaration of Helsinki. Approval was granted by the Ethics Committee of SMC Hospital (Date: September 17, 2024/003–2024). The committee waived the need for individual’s consent form due to the retrospective nature of the study and the inclusion of procedures conducted as part of the standard patient care. IRB decision was provided in writing via email. Patient records were anonymized prior to analysis, and the researchers did not have access to any information that could identify individual participants during or after data collection. Data were securely stored in compliance with institutional and international data protection standards, ensuring confidentiality and ethical adherence.

## Results

### Clinical outcomes

A total of 291 patients were randomly selected for this study, with 131 patients in 2022 and 160 patients in 2023. Table 1 presents the baseline characteristics of compliance percentages for each indicator, displaying the overall descriptive data and comparing rates between the two years. Key findings reveal significant improvements in drug-interaction consideration compliance, which rose from 93.8% in 2022 to 98.7% in 2023 (*P* = .047). Compliance with appropriate therapy duration increased markedly from 68.3% to 90.3% (*P* < .001), and culture follow-up interventions improved from 51.2% to 81.7% (*P* < .001). In contrast, compliance with the appropriate dose decreased from 89.3% in 2022 to 82% in 2023 (*P* = 0.040). Indicators such as appropriate indication, hypersensitivity avoidance, dose adjustment in renal impairment, and culture sensitivity testing showed no significant differences between the years. Median overall compliance also showed a significant increase, from 85.7 ± 38 in 2022–100 ± 33 in 2023 (*P* = .02), as illustrated in Table 1.

**Table 1:** Descriptive Data of Meropenem Compliance Indicators, 2022–2023.

Variables	Total (N = 279)	2022 (N = 124)	2023 (N = 155)	*P*-value ¥
Correct Indication	203 (70.0)	94 (71.8)	109 (68.1)	0.524
Absence of Hypersensitivity	286 (98.3)	129 (98.5)	157 (98.1)	0.393
Absence of Drug-interactions	280 (96.3)	123 (93.8)	157 (98.7)	**0.047** *
Correct Dose	240 (85.7)	117/130 (89.3)	123/150 (82.0)	**0.040** *
Dose Adjustment in Renal Impairment	66 (87.0)	21/24 (87.5)	44/52 (86.5)	0.437
Correct Duration	194 (79.3)	82/120 (68.3)	112/124 (90.3)	**<0.001** *
Culture Sensitivity Testing	189 (76.3)	66/87 (75.9)	123/160 (76.8)	0.858
Culture Follow-Up with Interventions	110 (66.4)	43/84 (52.2)	67/82 (81.7)	**0.001** *
% Overall Compliance ǂ (median ± IQR)	87.5 ± 33	85.7 ± 38	100 ± 33	**0.039** *

Frequencies not summing to 291 reflect Not Applicable Data (NA).

¥ Chi-square test was used to assess the relationship between variables across years for larger samples, and Fisher’s exact test was used for smaller samples.

ǂ Mann-Whitney U test was used to assess whether there was a difference in the distribution of overall compliance between the years.

* A *P*-value < .05 is considered statistically significant.

Upon assessing the difference in clinical outcomes between 2022 and 2023, clinical improvement within 72 hours increased from 90.4% in 2022 to 98% in 2023 (*P* = 0.017). Additionally, the percentage of patients transferred or discharged in a good clinical condition increased from 82.2% in 2022 to 95.7% in 2023 (*P* < 0.001), as shown in Table 2.

**Table 2:** Difference in patients’ outcomes between years 2022 and 2023.

Variables	2022 (N = 131)	2023 (N = 160)	Cramer’s V	*P*-value
	N (%)	N (%)		
Clinical Improvement within 72 hours (yes) ǂ	85/94 (90.4)	105/107 (98.1)	0.17	**0.017** *
	¥ NA = 37 (28.0)	¥ NA = 53 (33.0)		
Transfer/Discharge in a good clinical condition (yes) ǂ	83/101 (82.2)	132/138 (95.7)	0.23	**<0.001** *
	§ NA = 30 (22.9)	§ NA = 22 (13.8%)		

A chi-square test was used to assess the relationship between variables across years for larger samples, and Fisher’s exact test was used for smaller samples.

* A P < 0.05 is considered statistically significant.

ǂ The “Yes” percentage is calculated using only valid cases (i.e., excluding NA cases from the denominator).

¥ NA cases for the clinical improvement outcome represent patients who were not indicated to receive meropenem (e.g., patients undergoing operations who received meropenem due to fast turnover, making the outcome not assessable).

§ NA cases for transfer/discharge outcome represent chronic or long-term patients who were not transferred or discharged during the study period.

The Cochran-Armitage Trend Test was used to assess the progression of “clinical improvement within 72 hours” and “transfer/discharge in a good clinical condition” across consecutive quarters of 2022 and 2023. The analysis revealed a significant upward trend in both outcomes over time, with *P*-trend values of 0.003 and 0.001, respectively, indicating consistent improvement as the quarters progressed, as illustrated in Tables 3 and 4.

**Table 3:** Cochran-Armitage Trend test for “Clinical Improvement within 72 hours” outcome across the quarters of years 2022 and 2023.

Quarter-year		Clinical Improvement within 72 hours	
		N (%)	*P*-value
2022	**Q2_22** (N = 30)	25(83.3)	**0.003** *
	**Q3_22** (N = 31)	29(93.5)	
	**Q4_22** (N = 33)	31(93.9)	
2023	**Q1_23** (N = 36)	34(94.4)	
	**Q2_23** (N = 30)	30(100)	
	**Q3_23** (N = 24)	24(100)	
	**Q4_23** (N = 17)	17(100)	

Cochran-Armitage trend test was conducted to assess the trend in clinical improvement over the ordered quarters. P < 0.05 is considered statistically significant

Frequencies not summing to 291 reflect Not Applicable Data (NA). This includes patients who were not indicated to receive meropenem, such as those undergoing operations and receiving only 1 or 2 doses without indication and those with a fast turnover or who were discharged the next day, making the outcome not assessable.

* A *P *< 0.05 is considered statistically significant.

**Table 4:** Cochran-Armitage Trend test for “Transfer/Discharge” outcome across the quarters of years 2022 and 2023.

Quarter-year		Transfer/Discharge	
		N (%)	*P*-value
2022	**Q2_22** (N = 35)	25 (71.4)	**<0.001** *
	**Q3_22** (N = 34)	31 (91.2)	
	**Q4_22** (N = 32)	27 (84.4)	
2023	**Q1_23** (N = 36)	34 (94.4)	
	**Q2_23** (N = 37)	33 (89.2)	
	**Q3_23** (N = 39)	39 (100)	
	**Q4_23** (N = 26)	26 (100)	

The Cochran-Armitage trend test was conducted to assess the trend in “Transfer/Discharge” over the ordered quarters.

* A *P *< 0.05 is considered statistically significant.

Frequencies not summing to 291 reflect Not Applicable cases (NA). For the “Transfer/Discharge” outcome, NA cases refer to chronic or long-term patients who were not transferred or discharged during the study period.

In Table 5, the Mann-Whitney U test was used to assess differences in % overall compliance between patients with and without clinical improvement and transfer/discharge outcomes. In 2022, a significant difference was observed in % overall compliance between patients with and without clinical improvement (*P* = 0.002). Patients who showed clinical improvement had a higher median compliance (100, IQR = 36.7) compared to those without clinical improvement (85.7, IQR = 18.3). Similarly, a significant difference was observed for transfer/discharge outcomes in 2022 (*P* = 0.002). Patients with transfer/discharge had a higher median compliance (85.7, IQR = 40) compared to those without transfer/discharge (78.6, IQR = 40.7).

**Table 5:** Comparison of Overall Compliance by Clinical Improvement and Transfer/Discharge Outcomes (2022-2023).

Year	Outcome N (%)	Condition	Median (IQR) % Compliance	*P*-value
2022 (N = 131) ǂ	**Clinical Improvement within 72 hours** (Valid N = 85/94)	Yes	100 (36.7)	**0.002** *
		No	85.7 (18.3)	
	**Transfer/Discharge in a good clinical condition** (Valid N = 83/101)	Yes	85.7 (40.0)	**0.002** *
		No	78.6 (40.7)	
2023 (N = 160) ǂ	**Clinical Improvement within 72 hours** (Valid N = 105/107)	Yes	100 (50.0)	0.516
		No	100 (0)	
	**Transfer/Discharge in a good clinical condition** (Valid N = 132/138)	Yes	100 (40.0)	0.229
		No	100 (6.3)	

Mann-Whitney U test was used to assess differences in compliance ranks between the levels of clinical improvement within each year.

* A *P*-value < 0.05 is considered statistically significant.

ǂ The total N under each year includes all cases (Yes, No, and NA).

Valid cases refer to Yes and No cases used for percentage and median calculations.

NA cases for “Clinical Improvement within 72 hours” represent patients not indicated for meropenem, such as those undergoing operations and receiving only 1 or 2 doses without indication and those with a fast turnover or who were discharged the next day, making the outcome assessable.

NA cases for “Transfer/Discharge” refer to chronic or long-term patients who were not transferred or discharged during the study period.

In 2023, both clinical improvement and transfer/discharge outcomes showed uniform results with no statistically significant differences in % overall compliance. The median compliance for both outcomes was 100, with no significant variation between the groups (*P* > 0.05). These findings suggest a consistent pattern across both outcomes for 2023, with no substantial differences in compliance observed between the groups.

### Meropenem consumption and expenditure

Table 6 presents a significant decline in the DDD per 1000 patient-days across 2022 and 2023, as determined by one-way ANOVA (F-test = 4.693, p = 0.005). Post-hoc Bonferroni correction revealed the most substantial reduction between Q1 2022 and Q3 2023, with a 39% decrease from 230 to 140 DDD per 1000 patient-days. By Q4 2023, the DDD further declined to 121 per 1000 patient-days, marking a total reduction of 47% (p < 0.05 for all comparisons). Significant differences were observed between Q1 2022 and both Q3 and Q4 2023, indicating a meaningful shift in antimicrobial consumption patterns over time.

**Table 6:** Quarterly Trends in DDD per 1000 Patient-Days and Statistical Comparisons for 2022–2023.

Year	Quarters	DDD per 1000 patient-Day (Mean ± SD)	P-value	F-test	P-value (ANOVA)
2022	Q1	230 ± 46.24^a^^,^^b^	**0.025** *	**4.693**	**0.005** *
	Q2	176 ± 28.76	0.753		
	Q3	149 ± 31.91	0.056		
	Q4	154 ± 10.27	0.096		
2023	Q1	157 ± 2.80	0.131		
	Q2	190 ± 24.29	0.025		
	Q3	140 ± 20.33 ^a^	**0.025** *		
	Q4	121 ± 26.11^b^	**0.004** *		

^a^Quarter 3 is significantly different from Quarter 1 (p = 0.025, Bonferroni post-hoc test).

^b^Quarter 4 is significantly different from Quarter 1 (p = 0.004, Bonferroni post-hoc test).

* p < 0.05 indicates statistical significance.

**Fig 1:**
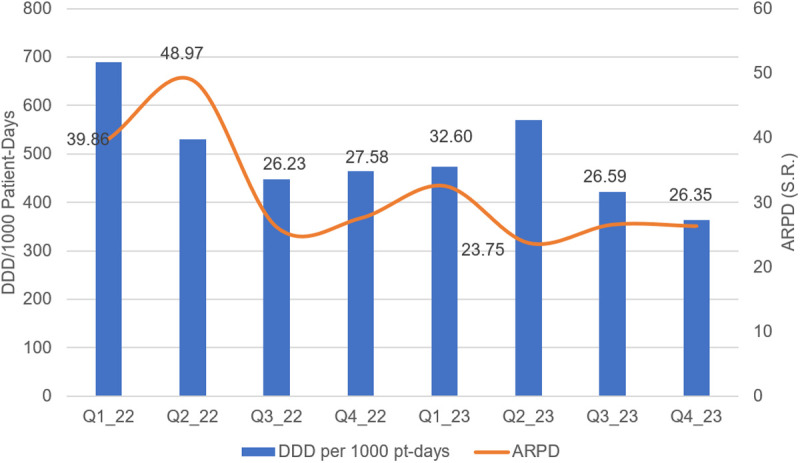
Meropenem Consumption Across Quarters of 2022-2023: Defined Daily Dose (Blue Bars) and Antimicrobial Cost per Patient Day (Red Line).

**Fig 2:**
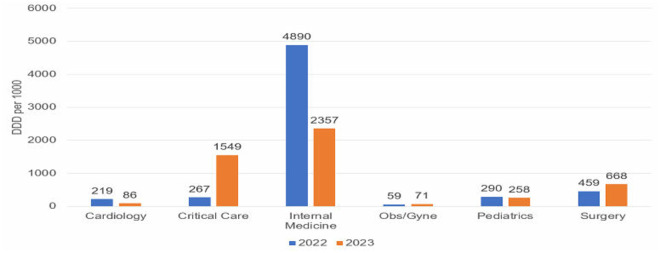
Pattern of meropenem prescription expressed as DDD per 1000 across medical specialties: 2022-2023.

As shown in Fig 1, total DDDs per 1,000 patient-days decreased by 47% from Q1 2022 to Q4 2023 (690–363 DDDs per 1,000 patient-days). During the same period, the total antimicrobial cost per patient-day dropped by 34% (39.86 to 26.35 ACPD). Meropenem consumption, measured in DDDs per 1,000 patient-days, was consistently highest in the internal medicine department, followed by the critical care department, across both 2022 and 2023, as illustrated in Fig 2.

The analysis of ACPD over 2022–2023 revealed a significant fluctuation in expenditure patterns. ACPD peaked in Q2-2022, showing a notable increase compared to Q1-2022 (p < 0.05), leading to higher antimicrobial costs. However, from Q3-2022 onward, ACPD consistently declined, with significant reductions observed in Q3-2022, Q2-2023, and Q4-2023 (p < 0.05). The most substantial decrease occurred in Q2-2023 and Q4-2023, where ACPD dropped by approximately 34–40% relative to Q1-2022 (39.855 S.R. to 26.352 S.R.), resulting in substantial financial savings as depicted in Table 7.

**Table 7:** Estimated Cost Savings and Expenditures During the Gradual Implementation of an AMS Program Across Quarters in 2022–2023.

Year	Quarter	Total Expenditure (S.R.)	Patient Days	ACPD (S.R.) ¥	Actual Cost Savings and expenditure compared with Q1_2022 ǂ
2022	Q1	62,173.66	4,815	39.855^a^	
	Q2	99,985.78	6,194	48.969^b, c, d, e, f^	−56,365.19
	Q3	64,525.16	7,406	26.226 ^a, c^	100,924.79
	Q4	64,148.92	6,964	27.584 ^e^	85,548.61
2023	Q1	74,025.22	6,802	32.597	49,306.20
	Q2	44,208.20	5,717	23.749^a, b^	92,162.28
	Q3	67,346.96	7,728	26.593^f^	102,694.78
	Q4	79,057.43	9,186	26.352^d^	123,648.55

¥ ACPD = antimicrobial cost per patient-day

ǂ Cost Savings Calculation: Each quarter’s ACPD was subtracted from Q1_2022 ACPD and multiplied by the corresponding number of patient-days. The total actual savings for Q2_2022–Q4_2023 amounted to S.R. 554,285, while the total additional expenditure was **S.R. 56,365.19**

A Kruskal-Wallis test with Bonferroni correction was conducted to compare ACPD ranks across different quarters. Quarters sharing the same letter indicate significant differences (p < 0.05).

## Discussion

### Clinical outcomes following ASP implementation

Effective ASP implementation is crucial in optimizing meropenem utilization and improving patient clinical outcomes. Our evaluation of meropenem use in the hospital revealed a significant improvement in the overall median compliance rate for meropenem, which increased from 85.7% in 2022 to 100% in 2023 (P = .02). This improvement underscores the high level of adherence to ASP guidelines, especially when compared to other studies reporting lower compliance rates and inappropriate prescribing practices [9,22]. Key factors contributing to this improvement included better drug-interaction considerations, appropriate therapy duration, and culture follow-up interventions. Specifically, compliance with drug-interaction considerations increased from 93.8% to 98.7% (P < .05), therapy duration improved from 68.3% to 90.3% (<0.001), and culture follow-up with interventions rose from 51.2% to 81.7% (P < 0.001). These results are consistent with prior studies showing that structured ASP interventions can effectively optimize therapy duration and culture follow-up practices [3,4]. In contrast, other studies have reported prolonged and unnecessary use of carbapenems, along with low acceptance rates for de-escalation and discontinuation interventions [9,22].

Despite the overall improvements, dosing compliance showed a significant decline in 2023 compared to 2022. This decline is attributed to the use of fixed doses for certain patient categories, such as those undergoing cystoscopy procedures and the early transfer/discharge before reaching the meropenem prescribing notification by the clinical pharmacist/ID consultant. Additionally, antimicrobial stewardship rounds were introduced only during Phase 3, which may have influenced this outcome. These findings highlight areas for targeted improvement in dosing practices to improve the practice gaps [23].

Clinical outcomes also improved significantly following ASP implementation. The proportion of patients showing clinical improvement within 72 hours increased from 90.4% in 2022 to 98% in 2023. Similarly, the percentage of patients discharged or transferred in good clinical condition rose from 82.2% to 92.7% during the same period. These findings are aligned with a systematic review highlighting the role of clinical pharmacists in optimizing antibiotic use and improving patient outcomes [24]. The observed improvements in clinical outcomes, particularly the rise in the clinical improvement rate within 72 hours, demonstrate that, when prescribed appropriately, meropenem is highly effective in managing severe infections. These results are consistent with previous studies showing similar positive outcomes with the timely and proper use of antibiotics [7,8].

Moreover, sequential improvements in clinical outcomes across quarters from 2022 to 2023 further emphasize the positive impact of ASP guideline implementation. These results align with research from Saudi Arabia, where phased implementation was also shown to improve patient safety and clinical outcomes. Such a step-by-step approach helps overcome physician resistance to changes in practice [20], ensuring better adherence and long-term success in antimicrobial stewardship.

Our study also found that in 2022, patients who demonstrated clinical improvement had significantly higher compliance rates (100%) compared to those who did not show improvement (85.7%; P = .002). Similar findings were observed for transfer/discharge outcomes, where patients discharged in good condition had higher compliance rates than those not discharged (85.7% vs. 78.6%; P = .002). In contrast, by the end of 2023, no significant differences in compliance rates were observed for these outcomes, reflecting the overall success of the ASP guidelines in improving clinical outcomes across all patient groups.

### Economic impact of ASP implementation

In addition to clinical improvements, our study also assessed the economic impact of the phased ASP implementation. We observed a significant 47% reduction in meropenem consumption from Q1-2022 to Q3-2023 (230 vs. 121 DDD per 1000 patient-days; P < 0.05), with consistent declines across successive quarters. Notably, meropenem consumption peaked in Q2-2022 before declining, which may be attributed to the fact that the antimicrobial stewardship program had only recently been initiated and was not yet fully implemented during that quarter. Restrictive measures on meropenem use were introduced in the second phase of the program (starting Q3-2022), which likely contributed to the subsequent reductions. Additionally, the early phase may have coincided with an increase in severe infection cases or empirical broad-spectrum prescribing practices prior to full enforcement of ASP protocols. These findings align with a multi-center study conducted in Saudi Arabia, which reported a 44% decrease in meropenem consumption over four consecutive years [19]. A recent systematic review and meta-analysis further supports these findings, showing a pooled reduction in meropenem consumption ranging from 6% to 55% following ASP implementation [25,26].

Our study observed a 40% reduction in ACPD by the end of Q4-2023, decreasing from 39.855 SR to 26.352 SR. Additionally, the cumulative cost savings were estimated at 554,285 SR, with low expenses projected at 56,365.194 SR. This reduction is in line with a large-scale study in Saudi Arabia, which observed a 33% decrease in ACPD after the first year of ASP implementation [19]. Furthermore, a systematic review encompassing 146 studies revealed that 92% of studies reported a reduction in antibiotic expenditures, as measured by antimicrobial cost per patient, following ASP implementation [27].

These findings underscore the economic benefits of phased ASP implementation in reducing both meropenem consumption and healthcare costs. By optimizing the use of meropenem, the hospital was able to significantly reduce overall antimicrobial expenditures, which may ultimately result in broader cost savings for the healthcare system.

## Limitations

This study has certain limitations that should be addressed. First, being a single-center investigation, the findings may not be fully generalizable to other healthcare settings. Additionally, demographic data such as patients’ age, gender, comorbidities, and socioeconomic factors were not included in the analysis, limiting the ability to assess their impact on compliance and clinical outcomes or to account for potential confounding factors. Furthermore, the observational design of the study does not establish a temporal causal relationship. Moreover, the findings on meropenem consumption and cost do not represent a true cost-benefit analysis; instead, they provide an overview of the program’s implementation, resource utilization, and observed benefits. Another important limitation is the lack of assessment of human factors, particularly compliance with the Antimicrobial Stewardship Program (AMSP) protocols and the implementation of corrective actions. These elements are critical in determining the program’s real-world effectiveness.

To address these limitations, future studies should involve multiple and diverse institutions and incorporate patients’ sociodemographic characteristics to enhance generalizability and provide a more comprehensive understanding of influencing factors. Additionally, applying methodologies such as interrupted time series analysis or propensity score matching could help establish stronger causal inferences. The cost-effectiveness analysis could be strengthened by incorporating incremental cost-effectiveness ratios (ICERs) and sensitivity analyses to assess economic outcomes under varying conditions. Future research should incorporate structured compliance monitoring, qualitative assessments, and staff feedback to better understand how behavioral and systemic factors influence AMSP outcomes. Despite these limitations, the study provides a strong foundation for successful guideline adherence and offers a model that other organizations can adapt and implement.

### Implications for future public health policies and economic planning

This study provides a foundation for integrating ASP interventions into national public health policies by demonstrating economic benefits and improved clinical outcomes. Future policies could standardize ASP implementation across healthcare facilities, allocate resources for continuous pharmacist-led ASP monitoring, and incentivize cost-effective prescribing. Economic planning should incorporate ASP-related training, real-time data monitoring systems, and periodic economic evaluations to sustain cost savings and enhance patient care.

## Conclusion

The phased implementation of ASP significantly optimized meropenem utilization, reduced antimicrobial costs, and improved prescribing practices. The improvements in patient clinical outcomes, including increased clinical improvement rates and higher transfer/discharge success, highlight the essential role of structured ASP interventions in enhancing patient care. Additionally, our study revealed a marked decline in meropenem consumption and cost, leading to substantial financial savings. Future studies should conduct comprehensive cost-effectiveness analysis to evaluate the economic implications and long-term impact of ASP on mortality and infection rates, providing valuable insights for policy makers and healthcare practitioners.
